# Mid‐term survival and physiological joint angles after double level osteotomy of severe varus osteoarthritis

**DOI:** 10.1002/ksa.12754

**Published:** 2025-07-13

**Authors:** Moritz Herbst, Steffen Schröter, Atesch Ateschrang, Christoph Ihle, Felix Finger, Tina Histing, Stefan Döbele, Cornelius Fischer, Marc‐Daniel Ahrend

**Affiliations:** ^1^ Department of Traumatology and Reconstructive Surgery, BG Trauma Center Tübingen Eberhard Karls University Tübingen Tübingen Germany; ^2^ Department of Traumatology and Reconstructive Surgery Diakonie Klinikum GmbH Jung‐Stilling‐Krankenhaus Siegen Germany; ^3^ Department of Orthopedics and Traumatology KSA Kantonspital Aarau Aarau Switzerland; ^4^ Department of Orthopedics m&i‐Fachkliniken Hohenurach Bad Urach Germany

**Keywords:** double level osteotomy (DLO), HTO, joint line obliquity, osteotomy

## Abstract

**Purpose:**

Double‐level osteotomy (DLO) is a joint‐preserving technique for the treatment of symptomatic varus knee osteoarthritis (OA) in cases of femoral and tibial combined deformity. The study aimed to investigate the mid‐term survival rate (>5 years) and restoration of postoperative joint angles.

**Methods:**

Sixty‐five knees underwent DLO (medial open wedge high tibial osteotomy (MOWHT) and lateral closing wedge distal femoral osteotomy (LCWDFO) between 2011 and 2015. Minimum follow‐up was 5 years. The survival rate was recorded and, in case of conversion to knee arthroplasty, the time of conversion. Radiographs were obtained preoperatively, 6 weeks postoperatively and at the last follow‐up. On radiographs mechanical tibiofemoral angle (mTFA), mechanical medial proximal tibia angle (mMPTA), mechanical lateral distal femur angle (mLDFA), joint line convergence angle (JLCA) and joint line obliquity (JLO) were measured. The clinical outcome was measured using International Knee Documentation Committee (IKDC), Oxford and Lysholm score.

**Results:**

Forty‐nine knees (75% follow‐up) were included after 8.0 ± 1.4 years. At the last follow‐up, six knees underwent arthroplasty (7‐year survival rate: 91.8%; 5‐year survival rate: 93.9%) in average after 5.1 ± 2.3 years. Preoperatively, there was a varus deformity of −10.0 ± 2.5° (mTFA). In addition, pathologic knee joint angles with an mMPTA of 84.7 ± 2.2°, an mLDFA of 91.4 ± 2.1°, an JLCA of 3.4 ± 1.8 and a JLO of 3.0 ± 1.9°. Six weeks postoperatively mTFA was 0.7 ± 2.2° with values of mMPTA 91.0 ± 2.3°, mLDFA 86.8 ± 2.0° and JLO of 2.8 ± 2.1°. In the mid‐term, a preserved leg axis (mTFA: −0.9 ± 2.7°) and preserved joint angles (mMPTA: 90.3 ± 2.7°, mLDFA: 87.1 ± 2.3°, JLCA: 4.3 ± 1.9 JLO: 3.2 ± 2.1°) were observed. At the last follow‐up, the IKDC, Oxford Knee Score (OKS) and Lysholm score were: 61.2%, 36.1 points and 78.3 points, respectively.

**Conclusion:**

The study demonstrates that DLO is an effective surgical technique to restore physiological joint angles in patients with severe preoperative deformity and symptomatic varus OA. Mid‐term results indicate good clinical outcomes and a low conversion rate to TKA.

**Level of Evidence:**

Level IV.

**Abbreviations**
DFO: distal femoral osteotomy
DLO: double level osteotomies
HTO: high tibial osteotomy
IKDC: International Knee Documentation Committee
JLCA: joint line convergence angle
JLO: joint line obliquity
KOOS: Knee Injury Osteoarthritis Outcome Score
LCWDFO: lateral closing wedge distal femoral osteotomy
mLDFA: mechanical lateral distal femur angle
MDF plate: medial distal femur plate
MHT plate: medial high tibia plate
MOWHTO: medial open wedge high tibial osteotomy
mMPTA: mechanical medial proximal tibia angle
mTFA: mechanical tibiofemoral angle
OKS: Oxford Knee Score
ROM: range of motion
SLO: single level osteotomy
TKA: total knee arthroplasty
UKA: unicompartmental knee arthroplasty

## INTRODUCTION

Osteotomies around the knee are a commonly used techniques to delay or avoid unicompartmental knee arthroplasty (UKA) in patients with symptomatic varus malalignment and medial osteoarthritis. Two main rationales emerged to develop and establish double level osteotomies (DLO) [4, 42].

One reason is that a pronounced malalignment is often based on a combined deformity of the distal femur and the proximal tibia [6, 14, 42]. In such cases, a single level osteotomy (SLO) often leading to a correction beyond the physiological limit of one joint partner due to compensation for the uncorrected other bone to achieve a neutral leg axis in the coronal plane [14].

The other reason is that postoperative unphysiological joint angles should be avoided, as they are associated with poorer clinical outcomes [24, 37, 43]. Due to these reasons, the DLO is used more frequently to postoperatively restore physiological joint angles in cases with extensive correction needed [3, 31, 42].

Over the years, the indications for a DLO have become increasingly homogenised [4] and were finally published as the ESSKA Consensus [10]. Currently, DLO is recommended in cases where a simulated high tibial osteotomy (HTO) results in an mechanical medial proximal tibia angle (mMPTA) of over 94° [3, 4, 19, 31, 42]. However, clinical and radiological mid and long‐term postoperative results following DLO with modern surgical techniques are limited.

This study aimed to determine the mid‐term survival rate of the DLO, as well as investigate the restoration of physiological joint angles and possible loss of correction during the postoperative follow‐up period. Additionally, the clinical‐functional outcome was assessed in the mid‐term. It was hypothesised that at least five years after DLO, only a small proportion of patients required a total knee arthroplasty (TKA). Radiologically, a shift in joint angles toward anatomical alignment was expected, even in cases of pronounced preoperative varus deformity.

## MATERIALS AND METHODS

For this retrospective study, all patients who had received a DLO between 2011 and 2015 were considered for study participation. 65 knees underwent DLO using the TomoFix™ plate (DePuy Synthes, Solothurn, Switzerland, medial distal femur [MDF] plate, of the contralateral side, for distal femur and medial high tibia [MHT] plate for proximal tibia respectively) with a varus deformity with a minimum follow‐up of 5 years.

**Figure 1:**
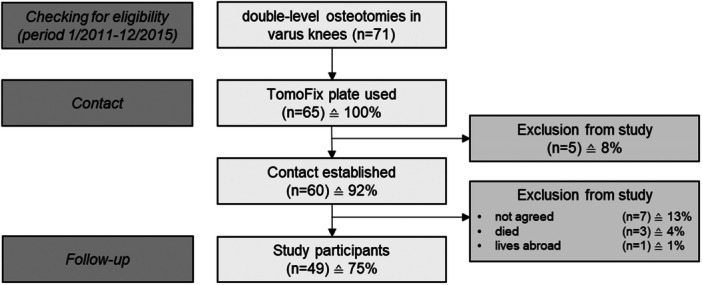
Study‐flowchart.

Five patients with no contact details were lost to follow up. Seven patients rejected study participation, three had died and one was living abroad. Forty‐one patients (49 knees) (follow‐up rate: 75%) were available for the follow‐up examination after an average of 8.0 ± 1.4 years (5.1–10.4) (Figure 1).

Patients' characteristics and demographic baseline data are shown in Table 1. For patients who received a DLO on both sides, there was at least a one‐year interval between both interventions. A positive vote was obtained from the local ethics committee (731/2020BO2) before beginning the study. Written informed consent has been obtained from all patients.

**Table 1:** Patient characteristics.

Number of patients	41
Number of knees	49
Sex	
Male	36 (73.5%)
Female	13 (26.5%)
Affected side	
Left	28 (57.1%)
Right	21 (42.9%)
Implant removal	42 (85.7%)
Follow‐up period [years]	8.0 ± 1.4 (5.1–10.4)
Age at surgery [years]	50.2 ± 7.4 (30.5–64.8)
BMI [kg/m²]	30.6 ± 5.6 (20.3–48.0)

*Note*: All values are presented as arithmetic means ± SD (minimum–maximum) or *n* (%).

Abbreviations: BMI, body mass index; SD, standard deviation.

### Indication and surgical technique

**Figure 2:**
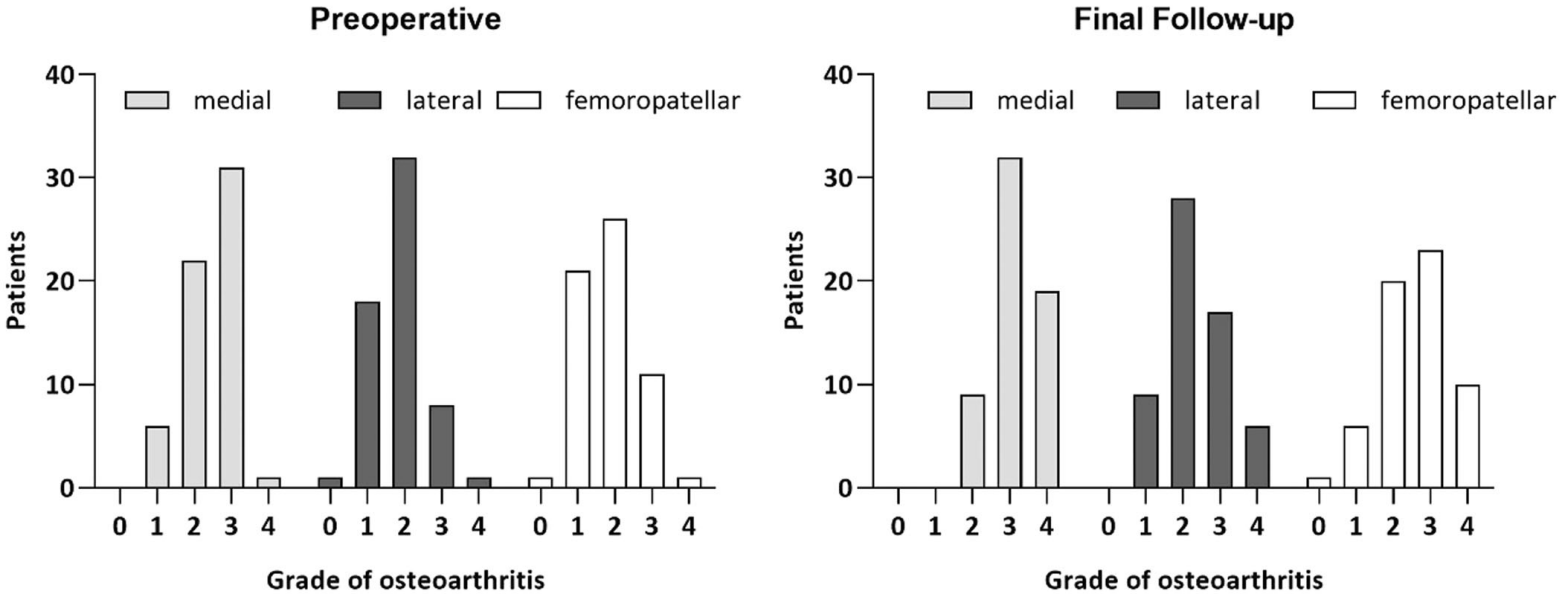
Distribution of osteoarthritis across knee joint compartments, preoperatively and at the time of follow‐up according to Kellgren and Lawrence.

The surgical indication for an osteotomy around the knee was given for symptomatic medial osteoarthritis with severe varus malalignment (Figure 2).

Patients were physically active and had no significant limitations in range of motion (ROM). The deformity analysis and osteotomy planning were performed using mediCAD® digital planning software (Hectec, Landshut, Germany) based on a full‐weight‐bearing, long‐leg radiograph. In the first step, an HTO was simulated. A DLO was applied if the HTO simulation yielded an mMPTA of > 94° or the deformity analysis showed a combination of lateral distal femur angle (mLDFA) > 90° and mMPTA < 87°. A slight overcorrection between 1° and 2° valgus was planned, depending on the degree of osteoarthritis in the medial and lateral compartments and the surgeon's preoperative decision‐making process. The surgical procedure is described in detail by Schröter et al. [42]. Briefly, summarised: Each DLO was preceded by a knee arthroscopy to confirm the indication and rule out additional pathologies. The DLO always began with the lateral closing wedge distal femoral osteotomy in biplanar technique [15] using the minimal subvastus approach [48]. Under protection of the neurovascular structures, the transverse incision was made, followed by the ascending incision. The osteotomy was closed slowly by applying valgus stress and was stabilised with the TomoFix™ MDF plate of the contralateral side. Additional compression was applied to the osteotomy using an eccentrically positioned bicortical screw in the combi‐hole, before the locking screws were placed. The new axial alignment was checked radiologically [42].

The medial open wedge HTO was performed as a biplanar osteotomy, as originally described by Staubli et al. [46] and modified by Lobenhoffer and Agneskirchner [28]. The osteotomy in the transverse plane was brought up to 1 cm to the lateral cortex, aimed to the tibiofibular joint tip. The biplanar ascending osteotomy was placed under the tibial tuberosity at an angle of 130°. The osteotomy was opened and stabilised using an anteromedial TomoFix™ MHT plate. A lag screw was used for posterior compression and later replaced with a locking screw. Finally, the alignment was checked again using an alignment rod [42].

Postoperative treatment included partial weight bearing of 20 kg on crutches for six weeks and consecutive, pain‐adapted weight bearing. No casts were used, and active physiotherapy was applied.

### Outcome parameter

The survival rate was defined as no conversion to knee arthroplasty (TKA) until the final follow‐up.

Radiographs of the knee joint were taken in two planes (anteroposterior and lateral view) and a long‐leg full‐weight‐bearing anteroposterior radiograph at the preoperative, 6‐week postoperative, and final follow‐up examinations (at least 5 years postoperatively). The radiological measurements were performed using the digital planning software mediCAD®. The mechanical tibiofemoral angle (mTFA), the mMPTA, the mLDFA, the joint line convergence angle (JLCA), and the JLO were recorded. The surgical accuracy was calculated [12, 41]. A more than 3° deviation from the planned result for the mTFA was considered as a relevant deviation from the intended surgical plan. In addition, the radiological grade of osteoarthritis of the individual knee joint compartments, medial, lateral, and femoropatellar compartments, was recorded according to Kellgren and Lawrence [23]. The clinical‐functional situation was assessed by clinical examination and using established clinical scores (International Knee Documentation Committee (IKDC), Knee Injury Osteoarthritis Outcome Score (KOOS), Oxford Knee Score (OKS), and Lysholm).

### Statistical analysis

Statistical analysis was performed using IBM® SPSS® Statistics Version 24 (Armonk, New York, United States) for Mac™OS (Cupertino, California, United States). The graphics were created with GraphPad Prism for Windows (GraphPad Software, Inc.; Version 8). Depending on whether a normal distribution was present, T‐tests or the Mann–Whitney *U*‐test were used for statistical analysis. For dependent variables, dependent T‐tests were calculated accordingly. A binary logistic regression analysis was performed to identify potential risk factors for conversion to TKA. Descriptive data is presented with the arithmetic mean ± standard deviation (minimum–maximum) or the median (minimum–maximum). The statistical significance level was set at *p* ≤ 0.05 for all tests.

## RESULTS

General data on the study population is shown in Table 1. Preoperative conventional X‐rays revealed, early‐stage osteoarthritis (Kellgren and Lawrence grading 1 and 2) in 20 and advanced osteoarthritis (Kellgren and Lawrence grading 3) in 28 cases for the medial knee joint compartment. The lateral and femoropatellar compartments showed early‐stage osteoarthritis. The progression of osteoarthritis was similar across all knee compartments over the follow‐up period (Figure 2).

### Survival and complications

At the last follow‐up examination (8.0 ± 1.3 years postoperatively, range 5.1–10.4), six knees were converted to TKA, resulting in a survival rate (no conversion to TKA) of 87.8%. All knees converted into a bicondylar knee arthroplasty without a higher degree of component coupling. Implantation occurred after an average of 5.1 ± 2.3 years (range 2.0–8.0). At earlier points during the follow‐up period, a 5‐year survival rate of 93.9% and a 7‐years survival rate of 91.8% were observed.

During the follow‐up period, five complications occurred. In two cases surgical intervention was required: One femoral hinge fracture treated with an additional medial plate after developing a pseudarthrosis six months postoperatively, and one postoperative seroma. Another femoral hinge fracture was already treated intraoperatively with an additional medial plate. Additionally, two postoperative deep vein thromboses were treated with temporary anticoagulation. None of the cases which had a complication were converted to a TKA during the follow‐up period.

### Radiological outcomes

Table 2 summarises the radiological preoperative deformity analysis, as well as the surgical plan and postoperative measurements. Preoperatively, all patients had a severe varus deformity with an average mTFA of −10.0 ± 2.5° (−15.8° to −5.8°). mLDFA (91.4° ± 2.1°, range 85.9°–98.0°) and mMPTA (84.7° ± 2.2°, range 79.7°–89.9°) showed non‐physiologic values, indicating a combined femoral and tibial varus deformity in most cases. Postoperatively, a neutral leg axis (mTFA: 0.7° ± 2.2°, range −5.9° to 4.5°) and physiological angles for mLDFA and mMPTA were achieved. During the follow‐up period, there was a significant re‐varisation of −1.7 ± 2.1 (0° to 8.2°) (*p* < 0.001) and an mTFA of −0.9° ± 2.7° (−7.5° to 4.0°) at the final follow‐up (Tables 2 and 3). The mMPTA showed a significant deviation of −0.9° (*p* = 0.021), and the mLDFA a deviation of 0.4° (*p* = 0.058). The JLCA was not significantly affected by the surgery; however, a significant increase was observed over the course of the follow‐up period (*p* = 0.044). The JLO remained constant with no statistically significant deviation (Table 3).

**Table 2:** Radiological parameters preoperatively, surgical plan and postoperatively.

		Total	Patient with TKA	Patients without TKA	*p* value
preOP	mTFA [°]	−10.0 ± 2.5 (−15.8 to −5.8)	−10.1 ± 2.6 (−14.0 to −7.5)	−10.0 ± 2.6 (−15.8 to −5.8)	0.964
	mLDFA [°]	91.4 ± 2.1 (85.9–98.0)	90.7 ± 1.5 (88.2–92.4)	91.4 ± 2.1 (85.9–98.0)	0.521
	mMPTA [°]	84.7 ± 2.2 (79.7–89.9)	84.2 ± 3.8 (79.7–89.8)	84.7 ± 1.9 (80.9–88.9)	0.749
	JLO [°]	3.0 ± 1.9 (0.3–6.9)	3.4 ± 2.5 (0.3–6.6)	2.9 ± 1.9 (0.3–6.9)	0.692
	JLCA [°]	3.4 ± 1.8 (0.2–9.4)	3.6 ± 1.1 (2.1–5.0)	3.3 ± 1.9 (0.2–9.4)	0.582
Plan	mTFA [°]	1.4 ± 0.7 (0.3–3.0)	1.1 ± 0.5 (0.3–2.0)	1.5 ± 0.7 (0.3–3.0)	0.133
	mLDFA [°]	86.4 ± 1.2 (83.3–89.0)	86.4 ± 1.1 (85.0–88.0)	86.4 ± 1.2 (83.3–89)	0.926
	mMPTA [°]	91.0 ± 1.2 (88.6–93.6)	91.0 ± 1.3 (89.1–93.1)	91.0 ± 1.2 (88.6–93.6)	0.951
	JLCA [°]	3.2 ± 1.8 (0.2–9.4)	3.6 ± 1.1 (2.1–5.0)	3.2 ± 1.8 (0.2–9.4)	0.394
postOP	Fem. wedge height [mm]	5.4 ± 2.0 (2–12)	4.7 ± 1.6 (3–7)	5.5 ± 2.0 (2–12)	0.336
	Tib. wedge height [mm]	7.9 ± 2.7 (3–13)	8.0 ± 3.7 (3 − 13)	7.9 ± 2.6 (4–13)	0.926
	mTFA [°]	0.7 ± 2.2 (−5.9 to 4.5)	0.2 ± 3.6 (−5.9 to 3.6)	0.7 ± 2.0 (−4 to 4.5)	0.963
	mLDFA [°]	86.8 ± 2.0 (82.2–91.0)	87.3 ± 1.4 (85.2–88.8)	86.7 ± 2.1 (82.2–91.0)	0.522
	mMPTA [°]	91.0 ± 2.3 (85.9–96.9)	90.6 ± 3.1 (85.9–94.1)	91.1 ± 2.3 (86.1–96.9)	0.963
	JLO [°]	2.8 ± 2.1 (0.1–8.1)	2.5 ± 2.5 (0.1–6.4)	2.8 ± 2.1 (0.3–8.1)	0.464
	JLCA [°]	3.7 ± 2.2 (0.2–11.2)	3.0 ± 1.0 (1.4–4.3)	3.8 ± 2.3 (0.2–11.2)	0.541
8 years	mTFA [°]	−0.9 ± 2.7 (−7.5 to 4.0)	−0.7 ± 4.1 (−5.8 to 4.0)^a^	−1.0 ± 2.5 (−7.5 to 2.5)	0.667
	mLDFA [°]	87.1 ± 2.3 (81.9–91.9)	87.7 ± 1.1 (86.2–89.2)^a^	87.0 ± 2.4 (81.9–91.9)	0.493
	mMPTA [°]	90.3 ± 2.7 (84.1–95.2)	90.3 ± 3.4 (85.5–95.1)^a^	90.3 ± 2.6 (84.1–95.2)	0.886
	JLO [°]	3.2 ± 2.1 (0.0–10.2)	3.4 ± 1.6 (1.6–5.2)^a^	3.2 ± 2.2 (0.0–10.2)	0.613
	JLCA [°]	4.3 ± 1.9 (0.0–9.1)	3.4 ± 1.7 (2.2–6.2)	4.4 ± 1.9 (0.0–9.1)	0.191

*Note*: All values are presented as arithmetic means ± SD (minimum–maximum).

Abbreviations: JLCA, joint line convergence angle; JLO, joint line obliquity; mLDFA, mechanical lateral distal femur angle; mMPTA, mechanical medial proximal tibia angle; mTFA, mechanical tibiofemoral angle; TKA, total knee arthroplasty.

^a^ The date refers to the last recording before conversion to arthroplasty.

**Table 3:** Axis deviations over the follow‐up period.

	postOP	Follow up	Difference	*p* value
mTFA [°]	0.7 ± 2.0	−1.0 ± 2.5	−1.7 ± 2.1	<0.001
mLDFA [°]	86.7 ± 2.2	87.0 ± 2.4	0.4 ± 1,2	0.058
mMPTA [°]	91.1 ± 2.3	90.3 ± 2.6	−0.9 ± 2.3	0.021
JLO [°]	2.8 ± 2.0	3.2 ± 2.2	0.4 ± 2.3	0.273
JLCA [°]	3.7 ± 2.2	4.3 ± 1.9	0.55 ± 1.8	0.044

*Note*: All values are presented as arithmetic means ± SD.

Abbreviations: JLCA, joint line convergence angle; JLO, joint line obliquity; mLDFA, mechanical lateral distal femur angle; mMPTA, mechanical medial proximal tibia angle; mTFA, mechanical tibiofemoral angle; SD, standard deviation.

### Surgical accuracy

Table 4 compares preoperative planning and postoperative results. Both the planning and postoperative results indicate a minor valgus overcorrection. The comparison shows high surgical precision without significant deviation for mLDFA and mMPTA. However, mTFA shows a small absolute but statistically significant deviation of −0.8 ± 2.1° between the planning and postoperative results (Table 4).

**Table 4:** Surgical accuracy.

	Planning	postOP	Surgical accuracy	*p* value
mTFA [°]	1.4 ± 0.7	0.7 ± 2.2	−0.8 ± 2.1	0.016
mMPTA [°]	91.0 ± 1.2	91.1 ± 2.3	0,0 ± 2.2	0.916
mLDFA [°]	86.4 ± 1.2	86.8 ± 2.0	0.3 ± 1.8	0.179

*Note*: All values are presented as arithmetic means ± SD.

Abbreviations: mLDFA, mechanical lateral distal femur angle; mMPTA, mechanical medial proximal tibia angle; mTFA, mechanical tibiofemoral angle; SD, standard deviation.

### Axis deviation and risk factor analysis of conversion to TKA

Eight patients had a leg axis deviation (mTFA) of ≥ 3° postoperatively compared to the preoperative plan. Two of these patients showed overcorrection, while six exhibited undercorrection, one of whom underwent conversion to TKA during the follow‐up period. The individual radiological values are listed in Table 5.

**Table 5:** Individual values of patients with an axis deviation (mTFA) of ≥ 3°.

	Patient	1	2	3	4	5	6	7	8	Total
preOP	mTFA [°]	−8.9	−8.2	−10.9	−13.1	−10.9	−12.2	−11.5	−9.9	−10.0
	mLDFA [°]	88.2	89.5	91.4	98.0	93.2	89.8	89.2	85.9	91.4
	mMPTA [°]	84.0	88.2	83.6	84.8	84.4	79.7	82.2	85.4	84.7
	JLCA [°]	4.7	7.0	3.2	0.2	2.1	2.1	4.5	9.4	3.4
Plan	mTFA [°]	0.5	1.0	1.6	1.0	2.0	1.0	1.1	0.3	1.4
	mLDFA [°]	85	84.1	86	89.0	87.0	86.0	85.7	83.3	86.4
	mMPTA [°]	90.2	92.1	90.8	89.8	91.1	89.1	91.2	93.0	91.0
	JLCA [°]	4.7	7.0	3.2	0.2	2.1	2.1	4.5	9.4	3.2
postOP	mTFA [°]	4.2	−4.0	−1.5	−2.0	−1.7	−5.9	4.5	−3.6	0.7
	mLDFA [°]	87.5	88.2	83.8	90.4	89.3	88.2	82.2	85.3	86.8
	mMPTA [°]	96.9	90.2	88.8	87.9	89.5	85.9	87.0	92.9	91.0
	JLCA [°]	5.1	6.0	6.5	0.5	1.9	3.5	0.3	11.2	2.8
	Coronal correction [°]	13.1	4.2	9.4	11.1	9.2	6.3	16	6.3	10.7
	Deviation [°]	+3.7°	−5.0	−3.1	−3.0	−3.7	−6.9	+3.4	−3.9	−0.8
Wedge	Femoral [mm]	5	5	6	11	8	5	4	2	5.4
	Tibial [mm]	9	4	8	6	10	13	11	8	7.9
	Complications	No	No	No	No	No	No	No	No	5
	Conversion	No	No	No	No	No	Yes	No	No	6

*Note*: Total values are presented as arithmetic means.

Abbreviations: JLCA, joint line convergence angle; mLDFA, mechanical lateral distal femur angle; mMPTA, mechanical medial proximal tibia angle; mTFA, mechanical tibiofemoral angle.

There was no statistically significant increased risk of conversion to TKA with a leg axis deviation of ≥ 3°. Furthermore, with an odds ratio of 0.728, there was no increased risk of conversion to TKA due to the postoperative deviation from the individual planning.

The extended analysis of risk factors for conversion to arthroplasty during the follow‐up, revealed no statistically significant risk factors among the examined parameters (Table 6). For female sex, a low predictive probability (significant Hosmer‐Lemeshow test) was observed, limiting the interpretability of odds ratio in this context (Table 6). The preoperative grade of osteoarthritis shows a tendency as a risk factor; however, only the femoro‐patellar osteoarthritis grade demonstrates statistical significance (Table 6).

**Table 6:** Risk factor analysis of potential risk factors for early conversion to TKA.

	Odds ratio	*p* value	Regressions‐coefficient	*p* value (Hosmer–Lemeshow test)
BMI	1.020	0.810	0.020	0.417
Age	1.023	0.689	0.023	0.413
Female	0.330	0.181	1.194	<0.001
PY	1.11	0.216	0.106	0.523
Med. osteoarthritis	−0.391	0.253	−0.940	0.709
Lat. osteoarthritis	0.646	0.553	−0.437	0.377
Fem.‐pat. osteoarthritis	0.287	0.040	−1.249	0.541
mLDFA preOP	1.205	0.415	0.187	0.444
mMPTA preOP	1.127	0.554	0.119	0.493
mTFA preOP	1.014	0.934	0.014	0.400
JLO preOP	0.876	0.559	−0.132	0.858
JLCA preOP	0.924	0.733	−0.079	0.215
Fem. wedge height	1.317	0.317	0.275	0.956
Tib. wedge height	0.984	0.921	−0.016	0.799
mLDFA postOP	0.856	0.492	−0.156	0.850
mMPTA postOP	1.107	0.592	0.101	0.245
mTFA postOP	1.113	0.580	0.107	0.132
JLO postOP	1.084	0.715	0.081	0.264
JLCA postOP	1.205	0.424	0.186	0.348

*Note*: All values are presented as arithmetic means ± SD.

Abbreviations: BMI, body mass index; JLCA, joint line convergence angle; JLO, joint line obliquity; mLDFA, mechanical lateral distal femur angle; mMPTA, mechanical medial proximal tibia angle; mTFA, mechanical tibiofemoral angle; PY, pack years; SD, standard deviation.

### Clinical‐functional results

**Figure 3:**
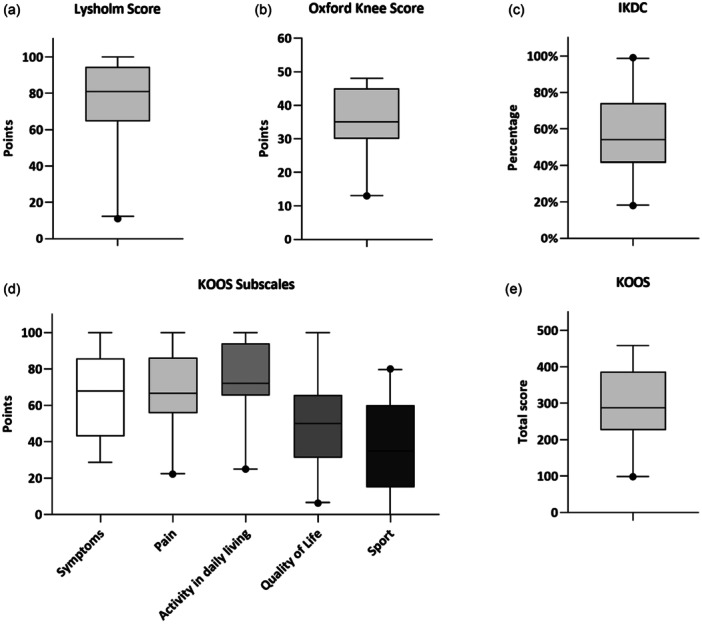
Boxplots: Results of clinical scores at the mid‐term follow‐up. (a) Lysholm Score results, (b) Oxford Knee Score results, (c) IKDC subjective results, (d) results of individual subscales, and (e) KOOS total score results. The upper and lower whiskers in the representation correspond to the 97.5th and 2.5th percentiles, respectively. IKDC International Knee Documentation Committee subjective score; KOOS, Knee Injury and Osteoarthritis Outcome Scores.

For all knees who did not receive TKA (*n* = 43), the clinical‐functional examination of the knee joint revealed no pathological findings regarding joint effusions, ligament stability, soft tissue conditions and range of motion. The results of the clinical scores are presented in Figure 3.

**Figure 4:**
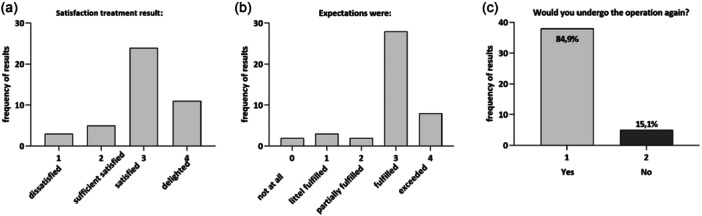
Bar charts fulfilment. (a) Satisfaction with treatment result, (b) fulfilment of treatment expectations, (c) undergo treatment again.

Figure 4 illustrates patient satisfaction and the extent to which their expectations were met.

## DISCUSSION

The most important finding was that DLO, as a treatment for severe symptomatic varus knee osteoarthritis, achieves a high mid‐term survival rate with physiological joint angles and good clinical function. Mid‐ to long‐term survival rates after DLO with modern indications and surgical techniques were previously unavailable. An observed survival rate of 87.8% after 8.0 ± 1.3 years in the present study is at least comparable to the known survival rates after HTO. A register data analysis from Finland described a 5‐year survival rate of 89% and a 10‐year survival rate of 73% [33]. Ahrend et al. reported a 5‐year survival rate of 91% and a 10‐year survival rate of 72.3%, based on three recent clinical cohort studies of HTO with a total combined cohort of 245 patients [2].

Thus, a more pronounced preoperative deformity, and the resulting greater surgical correction, does not appear to negatively impact survival rates following DLO. However, it should be noted that in the studies mentioned above, patients underwent HTO between 2005 and 2011 with varus deformities up to −19° [2]. According to current indication criteria, some of these cases would now warrant the use of a DLO [10].

The average time to conversion was 5.1 years, with a range between 2 and 8 years. In principle, a conversion within 5 years appears disproportionate and should be considered a failure. However, it must be taken into account that subjective treatment success is highly dependent on the individual patient's expectations [16]. Moreover, a positive effect of the joint angle normalisation achieved through DLO on a subsequent TKA is conceivable.

A patient‐specific indication strategy, considering preoperative deformity analysis and risk factors, is crucial for optimising outcomes and survival. Due to the lack of medium‐ and long‐term data following DLO, data on risk factors for early conversion to arthroplasty are also lacking. For HTO, factors such as advanced age, female sex, high BMI, and the severity of preoperative osteoarthritis as well as pronounced preoperative pain, are repeatedly discussed as risk factors [21, 22, 27, 36].

In the present analysis, no statistically significant risk factors for early conversion to TKA were identified for these parameters. However, there was a trend in the odds ratio for preoperative osteoarthritis severity (across all compartments) and for female sex, though this did not reach statistical significance (Table 6).

Moreover, the analysis of preoperative deformities revealed no statistically significant risk factors—neither with regard to the postoperative outcome nor to deviations from the individual surgical plan ≥ 3°. The reason for lacking statistical significance can be the small number of patients with conversion to TKA.

Reasons for a deviation of ≥ 3° from the preoperative plan could be measurement errors, primarily due to technically incorrect long‐leg radiographs [1]. Ultimately, intraoperative measurement inaccuracies [49], technical errors during surgery, or undetected hinge fractures with resulting loss of correction must also be considered as potential causes [39].

Recent studies have shown that the use of patient‐specific cutting guides can enhance surgical accuracy [29]. Additionally, measuring the osteotomy wedge angles appears to yield more accurate results than measuring the wedge height [49].

A two‐staged approach, involving a renewed analysis of the residual deformity followed by consecutive planning of the second osteotomy, could also enhance surgical accuracy. However, this would require rehospitalization and an additional period of partial weight‐bearing for the patient. A single‐stage DLO does not result in a significantly prolonged rehabilitation period in comparison to single level osteotomy [7]. Moreover, this decision in clinical practice depends on the respective healthcare system and the reimbursement policies for the procedures.

The modern indication for DLO, in cases of a simulated mMPTA > 94, resulted in postoperative physiological joint angles despite pronounced preoperative varus deformity. This could represent the biomechanical basis and explanation for the excellent survival rates observed.

The relevance and the impact on surgical outcome of physiological knee joint angles after osteotomy has been demonstrated in several recent studies [24, 37, 43]. A postoperative mMPTA > 95° has repeatedly been identified as a cause of reduced clinical outcomes [24, 37, 43]. An increased JLO has been shown to induce a significantly increased shear stress on the joint cartilage, even with a neutral leg axis in the coronal plane [32]. In addition, there is evidence of reduced clinical outcomes with JLO between 3° and 4° [9, 45]. Several studies have shown that, particularly in cases of pronounced varus deformity, HTO significantly increases the JLO compared to DLO [3]. In contrast, results after DLO have demonstrated physiological knee joint angles both postoperatively and during short‐term follow‐up in multiple studies [11]. These findings are consistent with the results of the present study and also indicates high precision and low error susceptibility of the procedure [3, 31, 35, 38, 42].

It should be noted that DLO involves a significantly more extensive preoperative deformity and mechanical correction as well as surgery on to bones. For this reason, some authors have suggested an increased complication rate for DLO in the past [20]. In this study, an overall complication rate of 10.2% was found, which is comparable to complication rates for a single level osteotomy. Miltenberger et al., in a systematic review, reported rates of 5.5% intraoperative and 10%–15% postoperative complications after HTO [30].

There was a slight re‐variation of −1.7 ± 2.1 (mTFA) and statistical significance for the change in mMPTA, mTFA and the JLCA during the follow‐up (Table 3). This indicates a combined revarization, involving both intra‐articular and extra‐articular components.

Generally, a loss of correction after osteotomy is considered a complication [41], but it is mostly discussed in relation to implant choice, hinge fractures, the filling of the osteotomy gap, or non‐union [5, 47]. Data regarding loss of correction over a longer follow‐up period are limited. For HTO, values between 2° and 3° are reported in the mid‐ to long‐term period [8, 34]. The present mid‐term results after DLO, with 1.7° axis deviation, thus show no increased risk of loss of correction despite significantly greater axis correction. The reason for this could be the reduced osteotomy wedge height of DLO, thereby reducing the risk of hinge fractures and delayed or non‐union healing of the osteotomy side [17, 25, 26, 44]. Through DLO, the individual correction angles of the osteotomy sites are significantly reduced. That also positively affects the soft tissue conditions, resulting in less ligament tension after osteotomy [42]. More balanced ligament conditions could even have a positive influence on a secondary TKA. The main problems of secondary TKA after HTO, in addition to the bony deformity of the proximal tibia, are tension imbalances in the capsular ligament apparatus [13]. Both disadvantages are reduced by DLO. Whether DLO actually provides better results than HTO in secondary TKA should be the subject of future research. At this point, the severe preoperative deformity before DLO should be reconsidered.

Clinically and functionally, the mid‐term results showed good outcomes comparable to other studies in this field [3, 31, 38, 42]. For example, the IKDC results of 58 ± 20% are comparable to Rupp et al. (66 ± 15.%) and Nakayama et al. (59 ± 12.6%) and slightly lower than Schröter et al. (77 ± 12%) [31, 38, 42]. The Lysholm score (76.9 ± 20.9), is higher than the results of Rupp et al. (73.1 ± 23.6) but lower than those of Schröter et al. (88 ± 13) and Akamatsu et al. (94.4 ± 6.3) [3, 38, 42]. These studies involve only short‐term follow‐up periods. A slight decrease of knee function postoperatively can be expected and was demonstrated in HTO surgery [18, 40]. Patients reach their highest knee function 12–18 months after HTO [18, 40].

## LIMITATIONS

This study employed a retrospective study design. Clinical scores are only available for the final follow‐up. A comparison to preoperative scores was not possible. However, radiological data are available for all time points and were evaluated. Due to the retrospective study design, a reduced follow‐up rate and selection bias cannot be ruled out. However, a follow‐up rate of 75% was achieved with an average follow‐up of eight years. Another study limitation is the lack of a comparison group with an alternative treatment method, such as single level osteotomy. Nevertheless, this research aimed to highlight the different indications and advantages. In the author's opinion, the procedures mentioned are not directly interchangeable with each other but rather complement each other regarding surgical treatment options for unicompartmental knee osteoarthritis depending on the present deformity. Eight patients received DLO on both knees. However, each knee was recorded separately, with at least one year between surgeries. The analysis of risk factors for conversion to TKA is based only on the six patients who received TKA during the follow‐up. Unfortunately, no more detailed analysis of the reasons for failure can be performed based on this dataset.

## CONCLUSION

This study demonstrates that DLO is an effective surgical technique to restore physiological joint angles in patients with severe preoperative deformity and symptomatic varus gonarthrosis. Mid‐term results indicate therapeutic success with good clinical‐functional outcomes and a very low conversion rate to TKA. The avoidance of an increased JLO and the maintenance of physiological joint angles throughout the follow‐up period likely represent the biomechanical basis for the clinical success of this treatment method. Further investigations into long‐term outcomes and the impact of DLO on secondary TKA are necessary in the future.

## AUTHOR CONTRIBUTIONS

**Moritz Herbst**: Data acquisition; statistical analysis; drafting and writing the manuscript. **Steffen Schröter**: Study design; revision of the manuscript. **Atesch Ateschrang**: Revision of the manuscript. **Christoph Ihle**: Revision of the manuscript. **Felix Finger**: Revision of the manuscript. **Stefan Döbele**: Revision of the manuscript. **Tina Histing**: Revision of the manuscript. **Cornelius Fischer**: Revision of the manuscript. **Marc‐Daniel Ahrend**: Study design; drafting and writing the manuscript; revision of the manuscript.

## CONFLICT OF INTEREST STATEMENT

Steffen Schröter is a member of the AO Joint Preservation and Osteotomy Expert Group. All other authors declare no conflicts of interest.

## ETHICS STATEMENT

The study was approved by the local ethics committee of the University of Tübingen (731/2020BO2). Informed consent was provided by all patients included into the study.

## Data Availability

The data sets generated during and/or analysed during the current study are available from the corresponding author on reasonable request.
